# Methods to Estimate the Between-Population Level Effective Reproductive Number for Infectious Disease Epidemics: Foot-And-Mouth Disease (FMD) in Vietnam

**DOI:** 10.1155/2024/4114217

**Published:** 2024-11-14

**Authors:** Umanga Gunasekera, Kimberly VanderWaal, Jonathan Arzt, Andres Perez

**Affiliations:** ^1^Veterinary Population Medicine, University of Minnesota, St Paul, Minnesota, USA; ^2^Foreign Animal Disease Research Unit, Plum Island Animal Disease Center, Greenport, New York, USA

## Abstract

Foot-and-mouth disease (FMD), which is endemic in 77% of countries globally, is a major threat to the global livestock industry. Knowledge of the reproductive number at the population level (i.e., farm level, herd level, or above) for FMD is important to estimate the magnitude of epidemics and design and implement effective control methods. Different methods, based on disparate assumptions and limitations, have been used interchangeably to compute and report reproductive numbers at the population level without a formal comparison between them. This study compares the results obtained when using alternative methods to compute between populations (*R*_bp_) for FMD using one single dataset collected over 10 years (2007–2017) at the commune-level swine farms in Vietnam. Seven spatial–temporal clusters were identified in the country, and the value of *R*_bp_ was computed on each of them using different analytical approaches, namely, epidemic doubling time, nearest neighbor, time-dependent reproductive number (TDR), sequential Bayesian (SB), and birth–death skyline (BDSKY) analysis in Bayesian evolutionary analysis by sampling trees 2 (BEAST2). Estimated *R*_bp_ values were relatively similar across methods ranging from 1.25 to 1.61. For the first time, the results here provide a comparison of different methods used to compute *R*_bp_ for FMD. Despite differences in assumptions and limitations, results suggest that different methods produce relatively similar outputs. Additionally, the results here provide foundational knowledge to support the evaluation and control of FMD epidemics in a population.

## 1. Introduction

Foot-and-mouth disease (FMD) is a contagious disease of cloven-hoofed animals caused by a FMD virus (FMDV) that imposes far-reaching losses to endemic and epidemic scenarios. FMD is endemic in many countries of Asia and Africa, and it is believed to still be present in regions of South America [1]. Countries free from FMD are under constant threat of disease introduction. FMD control is achieved through a combination of measures, including the use of vaccines and restrictions on the movement of infected and susceptible animals. Estimates on the pace of spread of the disease, such as the basic reproduction number (*R*0), are needed to quantify disease epidemic impact, plan control measures, and estimate the resources needed to control the disease (such as the number of vaccine doses or diagnostic tests required) [2].

*R*0 is the average number of secondary infections caused by one infectious individual in a fully susceptible population during its entire infectious period [3]. When we consider the reproduction number between farm level, herd level, or at a regional (such as commune or district) level, *R* may be defined as the average number of secondary farms/herds/regions infected by a single farm/herd/region, which in the literature has been indistinctly referred to as the farm-level (*R*_f_) or herd-level (*R*_h_) reproduction number. Here, we used the term *R*_bp_ to determine the reproductive number between populations at the commune level in Vietnam. According to the classification of the World Organization for Animal Health (WOAH), an epidemiological unit is a group of animals with a defined epidemiological relationship with approximately the same likelihood of exposure to a pathogenic agent. Such a similar likelihood of exposure may be because susceptible animals share a common environment. In Vietnam, the commune (referred to in other countries as a district) is used by the national veterinary services to report and record FMD outbreaks because it is considered the epidemiological unit for the country [4]. Hence, *R*_bp_ would be calculated at the commune level in Vietnam to evaluate the rate of transmission of the disease between populations in the country. Estimating the value of *R*_bp_ can help determine if disease spread is declining (*R* < 1) or expanding (*R* > 1) in an infected country. At the early stages of an epidemic, *R*_bp_ will increase when a population is mostly susceptible and will subsequently decrease when the number of susceptible individuals decreases.

Disparate analytical methods have been used to compute the value of *R*_bp_ at different population levels, and each of them is subject to different limitations and assumptions and requires different types of data. For example, analysis of disease incidence data or sequence data can be utilized to calculate R in a specified region [5]. These methods have been used interchangeably to estimate FMD spread, and, to the best of the authors' knowledge, the different methods have never been contrasted and discussed when applied to similar datasets from the same epidemiologic context. The objectives of this study were to compare the results obtained when *R*_bp_ was computed using different methods in the same dataset (swine FMD outbreak data), which was collected from Vietnam during 10 years. The study provides a comparative assessment of the methods used and discusses their assumptions and limitations for each approach.

## 2. Methods

### 2.1. Database and Analytical Approach

The dataset analyzed included data on the reported number of FMD outbreaks per month (Department of Animal Health, Vietnam), and FMDV VP1 sequences generated from various research and surveillance projects conducted in Vietnam [6–11]. Data were collected via passive surveillance at the commune level on a daily/weekly basis by the local sub-department of Animal Health [4]. Most cases were clinically diagnosed, and many cases were laboratory confirmed. An individual case was defined as an animal diagnosed as infected by local animal health professionals by the clinical signs only, and an outbreak was defined as an infected commune (WOAH, Terrestrial code) during the considered period. For swine, 43,662 cases were reported from 1,023 outbreaks (2007−2017) with a mean of 42.68 (SD 198) infected animals per outbreak. Each outbreak represents an infected commune and may include more than one farm. Because different livestock farms, including cattle, buffalo, swine, goats, and sheep, are distributed in the same geographical areas in Vietnam, swine farm clusters approximately overlap with other animal species FMD clusters [4].

Serotypes O and A are circulating in Vietnam, where FMD outbreak occurrence follows a cyclical pattern [4]. In total, 172 FMDV serotype O VP1 sequences were used from different provinces of Vietnam that are part of different spatial–temporal clusters identified in North and South Vietnam via a SatScan analysis. Sequence data were obtained from all three species swine, cattle, and buffaloes. Most sequences were collected from farms and slaughterhouses as part of active surveillance of clinical and subclinical bovines conducted by our collaborative team [11], whereas the remaining sequences were downloaded from GenBank (https://www.ncbi.nlm.nih.gov/genbank/). All Vietnam sequences used in the analysis included meta-data of location (to the province level) and date of sequence collection.

First, time–space clusters of swine outbreaks were identified through the study period in Vietnam using the permutation model of the scan statistic and, subsequently, *R*_bp_ was computed for each of the clusters using alternative methods.

### 2.2. Time-Space Cluster Detection

Space–time scan statistics are defined by a cylindrical window that moves across space and height defines the time (Kulldorff, Martin, 1997). Because no data were available regarding the background population at risk and because the objective of the analysis was to delimit temporally and spatially areas in which local transmission was likely to occur, we performed the space–time permutation model of the scan statistics. For this analysis, considering the temporal pattern of FMD across the period, height was adjusted to 6 months. The spatial dimensions were set to cover up to 50% of reported outbreaks. The moving cylinder across different spaces considers different geographical centroids in the entire country to detect the likelihood of clusters and the observed to expected ratio of reported outbreaks in identified clusters. The expected number would be the number of cases if there were no clustering. The statistical significance of the ratio was verified by Monte Carlo simulations (999 simulations).

### 2.3. Reproductive Number

The value of *R*_bp_ was computed for each of the detected time–space clusters at the commune level, using different analytical methods. The Friedman test was used to compare if variation within categories (cluster) was significantly different than that observed between categories (analytical method).

#### 2.3.1. Epidemic Doubling Time

Epidemic doubling time is a commonly used method to calculate *R*_bp_ [12, 13]. Epidemic doubling time calculates the time taken to double the cumulative incidence of cases. If the epidemic is growing at a constant rate, the doubling time also remains constant. Doubling time increases when the epidemic is in the declining phase. We have used epidemic doubling time *t*_d_ to calculate *R*_bp_ at the commune level for each cluster. Because case reporting dates vary substantially from the date of onset, the duration of infectiousness *D* was set to most likely (7 days), maximum (28 days), and minimum values (1 day). Epidemic doubling time was calculated throughout all spatial–temporal clusters, and the median *R*_bp_ value was obtained for each cluster considering *D* and *Td*.

Considering homogenous mixing and the exponential growth of the epidemic;

*R*_f _ = 1+$\frac{D}{t_{d}}$ ln2 [14]

• *D*—Duration of infectiousness, assumed as 1, 7, and 28 days.
• *t*_*d*_—Epidemic doubling time for reported outbreaks at communes (*t*_2–_*t*_1_).

#### 2.3.2. Epidemic Doubling Time (Stochastic)

We did not have information regarding the date of onset, but the date when an outbreak was reported from the communes and we had no information regarding the actual duration of infectiousness in each farm. Because of this, we performed the same calculations considering a pert distribution for the duration of infectiousness based on literature (3, 5, and 9) [13] and 1000 parametric bootstraps for a Poisson error structure around the harmonic mean doubling time td assuming homogenous mixing [15]. The harmonic mean was considered to account for the variability of the doubling time throughout the considered epidemic period. The *mc2d* R package was used for simulations [16]. This was performed for each cluster separately for the number of reported infected farms to compare with values from the initial doubling time method (2.3.1).

*R*_*f*⁣_ = 1+$\frac{D}{t_{d}}$ ln2

• *D*—Duration of infectiousness (Pert distribution).
• *t*_*d*_—Poisson error structure bootstrap based on median *t*_*d*_.

#### 2.3.3. Nearest Neighbor Infection Method

Given the semi-extensive backyard farming management system practiced in Vietnam, and 70% of the farmers are smallholder domestic farms, there is a strong possibility that fomites are shared and animals intermingle in neighboring farms (324 heads per km^2^ during the year 2017) [17] and therefore reported outbreaks at commune level may be acquired via the neighboring communes.

Reported outbreaks in each cluster were mapped using ArcGIS, and the resulting shapefile was imported to R. For each outbreak, outbreaks were reported in the next 7 days [18], and the distance between outbreaks was obtained. An outbreak at a neighbor commune was classified as the infection source to the next commune if they were closer in both the time and space of the initial reported outbreak. This process was repeated until all outbreak farms had a designated source except the very first outbreak reported in each cluster. Using the R package EpiContactTrace, the distribution and average number of outbreaks attributable to each source were calculated. An average *R*_bp_ value was obtained for each cluster.

#### 2.3.4. Time-Dependent Reproductive Number (TDR)

Originally used to calculate the R of severe acute respiratory syndrome (SARS), this method uses a likelihood-based procedure on who infected whom to estimate effective reproductive number based on the time of infection [19]. For this analysis, an epidemic curve is considered separately for each cluster at the temporal window identified in the Sat Scan analysis. We aggregated outbreaks reported by week to ensure every unit of time has at least one outbreak. This method was implemented via R package R0 [20]. Since this is a time-dependent method, the probability density function of the generation interval was obtained by the generation time function [21]. The generation interval is the time lag between the detection of first and secondary cases based on the clinical signs. The model assumes that the transmission occurred only between reported outbreaks and creates a directed network to calculate reproduction number averaging across all possible transmission networks. For the communes that reported outbreaks consecutively in time; it is more likely the commune that the first reported outbreak infects the next reported commune. The probability *p*_ij_ of case *i* at time *t* was infected by case *j* is given:

  $$\begin{array}{c} p_{ij}=\frac{N_{i}w\left(t_{i}-t_{j}\right)}{\sum_{i\neq k}N_{i}w\left(t_{i}-t_{k}\right)} \end{array}$$

*N* is the total number of cases and *w* is the generation time distribution. The *R*_bp_ for case *j* is $R_{j}=\sum_{i}p_{ij}$ and averaged over all outbreaks within the same date. Confidence intervals for each *R*_t_ value were obtained by 1000 simulations. We averaged different *R*_t_ values to obtain a single *R*_bp_ value for each cluster.

#### 2.3.5. Sequential Bayesian (SB) Method

This method also takes into account the epidemic curve for calculations considering only the exponential phase of the epidemic assuming random mixing [21]. In the SB method, the probabilistic formulation of SIR is integrated to show the number of cases at the next infection period *δt*. Following the SIR model, the total number of cases reported up to a time *T*(*t*) during an outbreak is given by:

  $$\begin{array}{c} \frac{dT\left(t\right)}{dt}=\beta \frac{S}{N}I \end{array}$$

Integrating this equation for *I* provides *R*_t_ for the next infection period time *δt* as a function of time. This is true only when the ratio of *S*/*N* is constant across time. In other words, where few cases are reported in a much larger population. By discretizing the differential equation to obtain the change in the total number of cases at δt, the following equation is used:

  $$\begin{array}{c} \Delta T\left(\delta t+t\right)=b\left(R_{t}\right)\Delta T\left(t\right), \end{array}$$

where *b* accounts for the infectious period of the disease and the number of infected up to time *δt* from the SIR model. This is true when an exponential growth of cases is reported, and the case number is lower compared to the total population. The parameter uncertainty of this model is estimated by Bayes' theorem. Sequential estimation of the initial reproductive number (*R*_t_) is carried out by using noninformative priors. A Poisson distribution is considered for reported case numbers. Bayes' theorem converts time series case numbers to a probability distribution as shown below, where N is the number of reported cases.

  $$\begin{array}{c} P\left(\left.R\right|N_{0},\ldots ,N_{t+1}\right)=\frac{P\left(N_{t+1}\left|R\right.,N_{0},\ldots ,N_{t}\right)P\left(\left.R\right|N_{0},\ldots .,N_{t}\right)}{P\left(N_{0},\ldots .,N_{t+1}\right)}. \end{array}$$

This process is described in detail in [21, 22]. This method was used to obtain *R*_t_ for each different time unit and was averaged for each cluster considering reported outbreaks at the commune-level swine farms.

#### 2.3.6. Bayesian Evolutionary Analysis by Sampling Trees 2 (BEAST2) Birth–Death Skyline (BDSKY) Serial Analysis

Bayesian evolutionary analysis tree provides a time scale for the emergence of FMDV sequences through time creating a maximum clade credibility tree. This tree determines the most recent common ancestor (MRCA) by calculating the coalescence of lineages through time [23]. Coalescent skyline plot introduced by Drummond et al. [24] accounts for changes in the virus population size (known as effective population size *N*_e_) considered in a piecewise fashion through time and jointly estimate *N*_e_ counting the number of coalescent events in each segment [24]. Based on the Wright–Fisher model, the coalescent rate is inversely proportional to the effective population size. Effective population size is the virus population that contributes progeny to the next generation of virus population. The effective population size *N*_e_ can be shown as below where *λ* is the coalescence rate [25];

  $$\begin{array}{c} \lambda =\frac{1}{N_{e}} \end{array}$$

The BDSKY model was developed assuming that the effective population size is stochastic and is used to calculate Re using assumptions related to a compartmental model [26].

Under the compartmental model assumptions, the effective population size *N*_e_ is considered inversely proportional to the transmission rate *λ*. This model focuses on forward-in-time disease transmission, and sequential sampling and accounts for sampling the population at fixed time points allowing several infections to be sampled at the same time. The sampling proportion is the fraction of sampled infections out of all infections. The considered total period is divided into intervals where the parameters change. This model considers the transmission rate parameter *λ*, with the assumption that each infected individual may transmit disease at a rate of *λ* and become uninfectious at a rate of *δ*. Once sampled, the infected individual is removed from the population meaning the individual is not transmitting the disease further. The transmission rate parameter *λ* is used to calculate Re through the well-known relationship Re = *λ*/*δ*. Re could be shown as

  $$\begin{array}{c} R_{e}=\;\frac{\lambda }{\delta }\; \end{array}$$

For sequence data collected over time considering the livestock population, the BEAST2 BDSKY serial model can be used to calculate *R*_bp_ using this approach. Based on the availability of sequence data, two spatial clusters were suitable to calculate *R*_bp_. A total of 105 sequences were available from all three species (cattle, swine, and buffalo) for cluster 1 geographical region and 43 of those were outbreak sequences. Outbreak sequences are defined as FMD sequences that were collected during an ongoing FMD outbreak from the farms. A total of 67 sequences were available from all three species for cluster 6 and 32 of those were isolated during outbreaks. Total sequences include both active and passive sampling, slaughterhouses, farms, and outbreak sequences. An initial analysis was performed to determine the population level reproductive number using total sequences for clusters 1 and 6. A secondary analysis was performed using only outbreak sequences available from clusters 1 and 6 to determine *R*_bp_ during an FMD outbreak.

All sequences were checked for recombination using Recombination Detection Program, version 5 (RDP4) and aligned after using Molecular Evolutionary Genetics Analysis, version X (MEGA X) [27], and identical sequences were removed. BDSKY serial method of BEAST2 was performed separately for clusters in both south (cluster 1) and north (cluster 6) using BEAST v2.6.3 [28]. BDSKY serial approach is based on the coalescent method where it is assumed a small random sample is collected from a larger infected population, which is the case for sequence data acquisition for FMD [26]. Tempest was used to detect the temporal signal using linear regression for root-to-tip distance and the sampling time [29]. bModel testing was used to select the best nucleotide substitution model for each dataset (Table 1) [30]. Since at a given time, we are considering sequence data from a single geographic cluster, which could be considered as a single epidemic for the considered serotype (Serotype O), a single substitution rate is assumed. Therefore, a strict molecular clock model was used [31]. Clock rate priors were determined from a previous BEAST1 analysis [11]. The origin time of the epidemic tree prior was determined using Tempest results and was assigned a log-normal distribution. For BDSKY serial analysis, a time when the index case was infected is earlier than the time to MRCA (tMRCA) [31]. Since Vietnam is endemic to FMD, Re prior distribution was set to one. Compared to the number infected, the number of sequences isolated is very few, sampling proportion priors were set to 1 in 9999 (beta distribution). Priors used for each parameter for each cluster are shown in Table 1. 100 million Markov chain Monte Carlo (MCMC) iterations were necessary to reach effective sample sizes of greater than 200. Tracer was used to analyze output, discarding 10% burnin [32]. Bayesian serial skyline plots were created for each cluster separately using the bdskytools R package [33]. The final maximum clade credibility tree was summarized using Tree Annotator after 10% burnin [28] and visualized using Fig Tree (http://tree.bio.ed.ac.uk/software/figtree/).

The data required for each method are shown in Table S1.

## 3. Results

Seven significant spatial–temporal clusters were detected, five in northern and two in southern Vietnam. A summary of the *R*_bp_ results per cluster estimated with each of the analytical approaches is shown in Table 2. The variation within categories (clusters) was not substantially different (*P* = 0.09) than between categories (analytical method), suggesting that the value of *R*_bp_ estimated for different clusters and using analytical methods were, in general terms, comparable to each other.

For the TDR method, the best-fitting distribution generation time interval was the Weibull distribution with a mean of 0.98 and a standard deviation of 0.03 weeks. For the SB method, the best-fitting generation time was log-normal distribution, with a mean of 2.81 and a standard deviation of 2.47 days. *R*_bp_ was not calculated for clusters 7 and 8 for TDR since there were more 0 s than case numbers once aggregated weekly. This analysis is sensitive for having 0 s in the dataset. Since the SB method was used as a comparison, the SB method was also not applied to clusters 7 and 8.

### 3.1. Results BDSKY Serial Analysis

Table 3 shows different parameters estimated using BDSKY serial analysis. BDSKY serial analysis provides an *R*_bp_ value for each cluster from the time of MRCA. In Table 3, median cluster *R*_bp_ values for the period where sequence data were available are displayed. Since the spatial clusters are considered, the sequences included in each cluster could belong to multiple phylogenetic clades which results in older MRCA. For cluster 1 outbreak sequences, the maximum clade credibility showed three different clades and tMRCA went back for 31 years. For cluster 1 outbreaks, (15/40) outbreak sequences were isolated from the 2011–2013 FMD outbreak period, in which the spatial–temporal cluster was identified. When all the sequences were considered for cluster 1 (population level *R*_bp_), tMRCA goes back for only 26 years contributing to a more recent sequence isolated during 2015–2019 via passive surveillance activities. For cluster 6 outbreak sequences, most of the sequences were (28/32) from the recent years of 2016–2019. FMD sequences in both clusters belonged to the Pan Asia lineage. In reality, the becoming uninfectious rate at the farm level is highly variable and complicated given the nature of FMD and may not represent a true scenario. Uncertainty of each estimate is shown with 95% highest posterior density (HPD) intervals.

**Figure 1:**
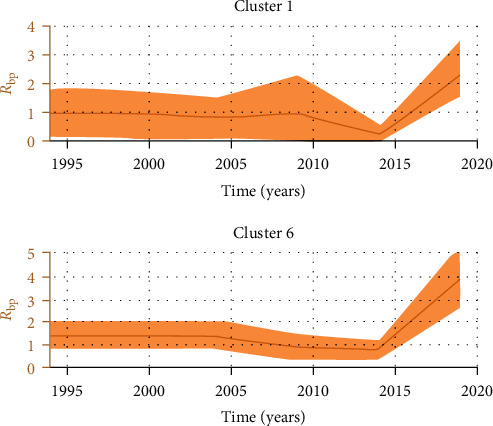
Birth–death skyline plots of clusters 1 and 6, using only outbreak FMDV sequences. The *y*-axis shows the inferred effective reproductive number parameter (*R*_bp_). The orange color shows the inferred Rbp over time. The *x*-axis shows time in years.

BDSKY plots of clusters 1 and 6 outbreaks are shown in Figure 1. For cluster 1 outbreaks, the *R*_bp_ value was calculated as closer to one (value of the prior) throughout the past where data were not available, hindering us from extrapolating *R*_bp_ values to the past. Because of this, in the table, we have shown the median *R*_bp_ values from the period where sequence data are available. *R*_bp_ values increase from 0.84 (mean 0.84, 95% HPD 0.07, 1.51) to 2.43 (mean 2.43, 95% HPD 1.58, 3.53) from 2010 to 2020. For cluster 6 outbreaks, *R*_bp_ values decreased from 1.41 (mean 1.41, 95% HPD 0.8–2.1) to 0.78 (mean 0.78, 95% HPD 0.3–1.16) during the period of 2010–2015 and then increases above 1–3.9 (mean 3.94, 95% HPD 2.6–5.3) 2015–2020. The median *R*_bp_ value for the whole period remained at 1.34 (1.39, 1.42). This is true for other clusters as the *R*_bp_ value would change across years and only the median *R*_bp_ value is shown for the period in Table 3. In endemic countries, FMD outbreaks tend to have a cyclic pattern throughout a few years. There are periods with no sequences available for a few years despite endemicity. However, there was an increase in sequence availability at the end of the analysis period of 2018 indicating the commencement of an outbreak from 2018 onward. This was found true compared to WOAH World Animal Helath Information System (WAHIS) reported FMD outbreak numbers from Vietnam during the same period.

## 4. Discussion

This study aimed to compare different methods used to calculate the commune level *R*_bp_ for FMD using a unique dataset obtained from Vietnam. Interestingly, and despite differences in the limitations and assumptions of the methods and the demographics of the country, the median of the mean *R*_bp_ values estimated per cluster was relatively similar across methods, ranging mostly between 1.1 and 1.6 (Table 2). Except for the Bayesian skyline method, all the other methods are based on reported swine FMD outbreak numbers at the commune level in Vietnam and the date of the outbreak. We mainly focused on using different methods of *R*_bp_ calculation considering the swine population, which is not fully representative of FMD circulation in Vietnam. Epidemic doubling time, nearest infectious neighbor, and TDR methods are efficient—when outbreak data are available from surveillance activities and population data are not available. Whereas, most interestingly, sequence data-based methods produced comparable results for identified clusters when a sequence database exists without access to the outbreak or spatial data. Additionally, different methods were associated with disparate limitations and assumptions, which make them suitable for differential availability of data.

We preferred to estimate the parameters only for closely located outbreaks because of the higher chances of a relationship between outbreaks. Epidemic doubling time methods are greatly affected by the number of reported outbreaks that change with a particular area's surveillance activities and available data collection funding. The highest *R*_bp_ value, omitting the value from the TDR method for cluster 1 (14.34, 95% confidence interval (CI) = 10.46–18.18), was estimated from the stochastic epidemic doubling time method, where a range of values was tested for the duration of infection. However, the confidence interval of this value reaches infinity indicating lower precision. Stochastic epidemic doubling time provides better results as it allows adjusting for different durations of infection, especially because the time to report an outbreak may vary from region to region, and there was no information regarding the onset of an outbreak and no records of how long farms were infected. In the nearest infectious neighbor method, for a 6-month outbreak window, we only considered up to 7 days for one farm to infect another farm which could be a limitation.

According to the literature, the TDR method is appropriate for acute infections where disease incidence is frequently reported. This model does not account for contact heterogeneity within clusters and does not consider who infected whom. For this analysis, outbreak numbers were aggregated weekly to create an epidemic curve. Although FMD outbreaks occur daily, and although FMD is a reportable disease for WOAH, official reports can occur weekly/monthly based on the quality of the surveillance system in endemic countries. This could be a bias identified in this study since we used reported outbreak data. For example in Cluster 1, during the considered period, there were 2 weeks with unusually high numbers of reported outbreaks that resulted in a higher *R*_bp_ compared to any other *R*_bp_ value for cluster 1. SB method is identified as a comparatively better method than the TDR method since it accounts for past outbreak trend by a probability distribution [21].

One important limitation of these methods is that there is evidence that FMDV can move from one region to the other due to animal movement, and a similar lineage of FMDV was detected in both South and North Vietnam during the same period of outbreaks [11]. Therefore, infection can be acquired from other methods apart from neighboring farm infections. There have been discussions on the validity of phylodynamics in public health policy [34]. However, our study supports that compared to the other methods, we can obtain approximately similar values when applied to outbreak sequence data. With the rising availability of pathogen sequence data, these approaches appear to produce similar results to other methods for quantifying epidemiological parameters [10, 35, 36]. In the coalescent approach, a phylogenetic tree is used to infer the transmission history of pathogens. When phylogenetic data are considered, there are many obstacles to inferring epidemiological parameters related to transmission. For RNA viruses like FMDV, it can be difficult to determine which host transmitted to which host based solely on sequence variation [37] or in our case which farm/herd transmitted to which farm/herd in a given spatial cluster. Also, the whole infected population is rarely sampled to obtain sequence data. There are surely missing sequences that should be accounted for in the transmission process. Improvements are seen in methods such as structured coalescent transmission tree inference (SCOTTI) [38] and the BDSKY method that accounts for undersampling.

*R*_bp_ values obtained via the BDSKY method provide a median value for a range of years starting from the tree's root (1994 for serotype O). This value is not directly comparable with the values obtained from the other methods that consider a shorter time duration. For comparison purposes, median *R*_bp_ values of outbreaks where sequence data were available are included in the analysis for clusters 1 and 6. If we look at the comparable values for the period of spatial–temporal clusters, for example, for cluster 1 during the period of 2011, the *R*_bp_ value was close to 0.85, and for cluster 6, during 2011, it is around 1. These point values are compatible with *R*_bp_ values from other methods. Since serotype O is most common in Vietnam, only outbreak sequences from serotype O were considered for BDSKY analysis. Due to the limited number of available FMDV sequence data from Vietnam, we have used sequence data from all three species (cattle, buffalo, and swine) to calculate the effective reproductive number. This is a limitation of this study. However, the *R*_bp_ values approximately overlap with the other methods.

*R*_bp_ values obtained from the BDSKY method, when applied at the population level, show values closer to one indicating that FMD is endemic in Vietnam. FMDV sequences obtained from farms, slaughterhouses, and outbreaks in combination represent virus circulation at the cluster level. When outbreak sequences alone are considered at each cluster level, we obtained *R*_bp_ values above 1, indicating the propagation of outbreaks. Yet the longer MRCA values for each cluster indicate that multiple clades are circulating within one geographic cluster. Outbreak sequences considered in this analysis for each clusters 1 and 6 belong to the Pan Asia lineage. There is enough diversity within a lineage that all sequences circulating within the country belonging to a single lineage are not part of the same circulation event. We do not have enough sequence data from each different strain/sublineage level to run these analyses separately for Vietnam. Ideally, a sensitivity analysis for the BDSKY method may be performed using a subset of sequence data from the outbreak sequences and the same analysis to see whether we can obtain similar R values from the subset of data [5]. However, in this study, there were not enough outbreak sequences available for each cluster-level region for that kind of analysis.

Since host susceptibility is inconsistent, and also influenced by external factors, such as vaccination, status of natural immunity, or population density, it is virtually impossible to have reasonable expectations for the value of *R*_bp_ for FMD without considering the specific epidemiological scenario and, in terms of mathematical modeling, make more sense than assuming a broad representation of FMD as a whole, which may be too variable and uncertain to be able to be modeled. Since different models provide different *R*_bp_ values, it is best to validate *R*_bp_ calculation methods. We obtained similar median values of *R*_bp_ for different clusters utilizing different methods (*R*_bp_ = 1.1–1.6). This value is closer to the farm-level *R*_bp_value expected for an FMD endemic country (*R*_bp_ = 1) and lower than observed in FMD epidemic situations [39–42]. Methods used here are applicable at other FMD endemic settings indicating that different types of available data can be used to obtain comparable results in an endemic setting that in turn support the design and evaluation of control programs.

In Vietnam, a national FMD control program is in place that includes vaccination and post vaccine monitoring. The locations most densely populated by all three species of livestock (cattle, buffaloes, and pigs) that are susceptible to FMD are those where the spatial temporal FMD clusters were detected in this analysis, which is consistent with a relatively simple dynamics of an endemic disease. Because Vietnam borders are porous, frequent FMDV incursions are observed from neighboring countries. The R value calculated here (*R*_bp_ = 1.1–1.6) may reflect a status of dynamic equilibrium, in which control actions (such as vaccination and post vaccine monitoring) help to mitigate disease impact, but are yet insufficient to eliminate the disease.

## 5. Conclusions

The results here suggest that, in general terms, the alternative methods used in the literature to compute *R*_bp_ may lead to estimates that are, in general, consistent across methods, which facilitates the comparison between studies, and, most importantly, increase the opportunities for adjusting the methods to the data available in the affected country. These results contribute to understanding the epidemiological dynamics of FMD spread and will ultimately help the formulation of models intended to evaluate measures for disease prevention and control globally.

## Figures and Tables

**Table 1:** Priors used in each cluster for sampling, clock rate, effective reproductive number Re, becoming uninfectious rate, and the selected nucleotide model for each cluster for BDSKY analysis.

Parameter	Cluster 1	Cluster 6
Sampling proportion	1 in 9999 is sampled beta distribution	1 in 9999 is sampled beta distribution
Nucleotide model	JC69	TN93
Strict clock rate	0.0078	0.0078
Re prior	Log normal (median 1)	Log normal (median 1)
Becoming uninfectious rate	14 days Log normal	14 days Log normal

Abbreviation: BDSKY, birth–death skyline.

**Table 2:** Summary of *R*_bp_ values estimated using alternative analytical methods for different spatial–temporal clusters of FMD detected in Vietnam.

Cluster (number of cases, radius (km))	Date	Doubling time *R*_bp_	Doubling time stochastic *R*_bp_	Nearest infectious neighbor *R*_bp_	TDR *R*_bp_	SB *R*_bp_	BDSKY method *R*_bp_
Cluster 1 south (68, 28.66)	January 17, 2011–March 1, 2011	2.45	3.53 (1.46-inf)	1.91 (1.09–2.73)	14.34 (10.46–18.18)	1.68 (0.92–2.92)	1.17 (0.68, 1.55)

Cluster 2 south (82, 260.73)	January 6, 2007–May 5, 2007	1.6	1.8 (1.18–3.73)	2.44 (1.66–3.22)	1.12 (0.78–1.5)	1.11 (0.26–1.98)	—

Cluster 3 north (29,36.7)	November 23, 2010–May 27, 2011	1.17	1.27 (1.06–1.73)	1.25 (0.89–1.61)	1.24 (0.74–1.76)	1.89 (0.22–5.83)	—

Cluster 5 north (15, 81.69)	January 22, 2012–March 4, 2012	1.87	2.18 (1.26–5.99)	1.25 (0.56–1.93)	1.61 (0.67–2.87)	1.12 (0.05–1.94)	—

Cluster 6 north (52, 80.26)	October 17, 2010–March 12, 2011	1.23	1.44 (1.09–2.01)	1.6 (1.38–1.82)	1.61 (0.67–2.87)	1.11 (0.21–2.06)	1.34 (0.59, 1.99)

Cluster 7 north (15, 95.63)	September 12, 2009–February 28, 2010	1.11	1.13 (1.08–1.3)	1.33 (0.16–2.5)	—	—	—

Cluster 8 north (11, 80.96)	March 9, 2013–May 10, 2013	1.29	1.43 (1.11–2.1)	1.4 (0.8–1.99)	—	—	—

Median point value	—	**1.41**	**1.44**	**1.4**	**1.61**	**1.12**	**1.25**

*Note:* The location, number of farms, and the radius of each cluster are shown in the cluster column. The bold indicates a main summary value obtained from the different methods across different clusters.

Abbreviations: BDSKY, birth–death skyline; FMD, foot-and-mouth disease; SB, sequential Bayesian; TDR, time-dependent reproductive number.

**Table 3:** Parameter posterior values and 95% HPD intervals estimated by BDSKY serial analysis.

Parameter	Cluster 1	Cluster 1OB	Cluster 6	Cluster 6 OB
Sampling proportion	0.009 (0.003–0.015)	0.038 (0.002, 0.114)	0.0009 (0.0003, 0.001)	0.0009 (0.0004, 0.001)
tMRCA years	25.75 (21.57, 31.16)	31.40 (27.34, 34.42)	19.81 (16.37, 23.63)	13.61 (12.07, 15.27)
Strict clock rate subs/sites/year	0.007 (0.005, 0.009)	0.0054 (0.004, 0.006)	0.011 (0.009, 0.012)	0.0026 (0.002, 0.003)
Becoming uninfectious rate (duration of infection at the cluster level)	1/4.329 (3 months)	1/1.047 (11 months)	1/18.14 (18 days)	1/5.16 (2.4 months)
*R*_bp_ value	1.04 (0.95, 1.09)	1.17 (0.68, 1.55)	1.07 (0.98, 1.05)	1.34 (1.39, 1.42)

Abbreviations: BDSKY, birth–death skyline; HPD, highest posterior density; OB, outbreak; tMRCA, time to most recent common ancestor.

## Data Availability

Data were derived from the following resources available in the public domain: https://doi.org/10.3390/v13112203, viruses; https://doi.org/10.1111/tbed.13370, transboundary and emerging diseases; and https://doi.org/10.3390/v15020388, viruses.

## Supporting Information

Supporting Information Table S1 Data used for each method when calculating *R*_bp_ in the identified spatial–temporal clusters.
