# Supplementary material for: Methods to Estimate the Between-Population Level Effective Reproductive Number for Infectious Disease Epidemics: Foot-And-Mouth Disease (FMD) in Vietnam
Source: Transbound Emerg Dis. 2024 Nov 14;2024:4114217. doi: 10.1155/2024/4114217 (PMC12016952; doi:10.1155/2024/4114217)
Supplement: Supporting Information — Table S1 Data used for each method when calculating Rbp in the identified spatial–temporal clusters. [file 4114217.f1.docx]

Supplementary table 1

Data used for each method when calculating R_bp_ in the identified spatial-temporal clusters.

| Method | Type of data | Location data |
| --- | --- | --- |
| Epidemic doubling time | Duration of infectiousness  Epidemic doubling time for reported outbreaks at communes | Not required |
| Epidemic Doubling time (stochastic) | Duration of infectiousness  Epidemic doubling time for reported outbreaks at communes | Not required |
| Nearest Neighbor Infection Method | Closer in time outbreak location | Closer in space outbreak location |
| Time-dependent reproductive number (TDR) | Weekly aggregated reported outbreaks consecutively in time | Not required |
| Sequential Bayesian method (SB) | Reported outbreak numbers consecutively in time | Not required |
| BEAST2 Birth Death Skyline serial analysis | Sequence data with time of sampling | Sequence data location of sampling |
